# Simultaneous 18F-FDG PET/MRI in tuberculous spondylitis: an independent method for assessing therapeutic response - case series

**DOI:** 10.1186/s12879-019-4469-2

**Published:** 2019-10-15

**Authors:** Ikchan Jeon, Eunjung Kong, Sang Woo Kim

**Affiliations:** 1 Department of Neurosurgery, Yeungnam University Hospital, Yeungnam University College of Medicine, 170, Hyeonchung street, Nam-Gu, Daegu, 42415 South Korea; 2 Department of Nuclear Medicine, Yeungnam University Hospital, Yeungnam University College of Medicine, Daegu, South Korea

**Keywords:** 18F-FDG PET, Tuberculous spondylitis, MRI, Therapeutic response

## Abstract

**Background:**

^18^F-fluorodeoxyglucose positron emission tomography (18F-FDG PET) shows great potential for diagnosis and assessing therapeutic response of tuberculous spondylitis. Tuberculous spondylitis required long-term anti-tuberculosis (TB) medication therapy, and the optimal duration of therapy is controversial. There is still no clear way to tell when the anti-TB therapy can safely be discontinued.

**Case presentation:**

Three patients with tuberculous spondylitis were evaluated for therapeutic response using 18F-FDG PET/magnetic resonance imaging (MRI). Clinical and hematological improvements were achieved after about 12 months of anti-TB medication therapy, and we considered whether to discontinue the therapy. There was no relapse during one year of follow-up after discontinuation of 12 months anti-TB medication based on the low maximum standardized uptake value (SUV_max_) of 1.83 in one patient. However, the other two patients continued further anti-TB medication therapy based on the high SUVmax of 4.14 and 7.02, which were suspected to indicate active residual lesions in the abscess or granulation tissues. Continuous TB was confirmed by the bacterial and histological examinations.

**Conclusions:**

18F-FDG PET/MRI has metabolic and anatomical advantages for assessing therapeutic response in TB spondylitis, and can be considered as a helpful independent and alternative method for determining the appropriate time to discontinue anti-TB medication.

## Background

Skeletal tuberculosis (TB) accounts for about 10–20% of extra-pulmonary TB, which comprises about 2% of total TB cases [1, 2]. The most common form of skeletal TB is tuberculous spondylitis, which accounts for about 50% of all cases of skeletal TB [3]. In most cases, patients present pain as a main symptom. It is difficult to diagnose tuberculous spondylitis quickly because the symptoms are ambiguous. Tuberculous spondylitis commonly develops in the thoracolumbar region and is often accompanied by paravertebral or epidural abscesses [4].

As of yet, there is no definitive guideline for treatment period and regimen, and also no clear assessing modality for the therapeutic response. Several studies have recently been reported on the role of ^18^F-fluorodeoxyglucose positron emission tomography (18F-FDG PET) for diagnosis and assessing therapeutic response of tuberculous spondylitis [5, 6]. However, the results of the 18F-FDG PET study associated with the actual timing of the discontinuation of anti-TB medication have not been confirmed. In this paper, three patients diagnosed with tuberculous spondylitis were evaluated for therapeutic response using simultaneous 18F-FDG PET/magnetic resonance imaging (PET/MRI) after 12 months of anti-TB medication therapy, and the associated clinical and radiological findings were analyzed and compared with the results of the existing literatures.

## Case presentation

The first patient was a 43-year-old woman who had been suffering from back pain for about eight months. Infectious spondylitis on L2–3 with pre- and intra-vertebral abscesses was suspected from the MRI. The blood inflammatory indexes including C-reactive protein (CRP) and erythrocyte sedimentation rate (ESR) were increased by 1.57 mg/dL and 88 mm/h from the initial 0.7 mg/dL and 30 mm/h, respectively (normal ranges are defined as < 0.5 mg/dL for CRP and < 20 mm/h for ESR).

**Fig. 1:**
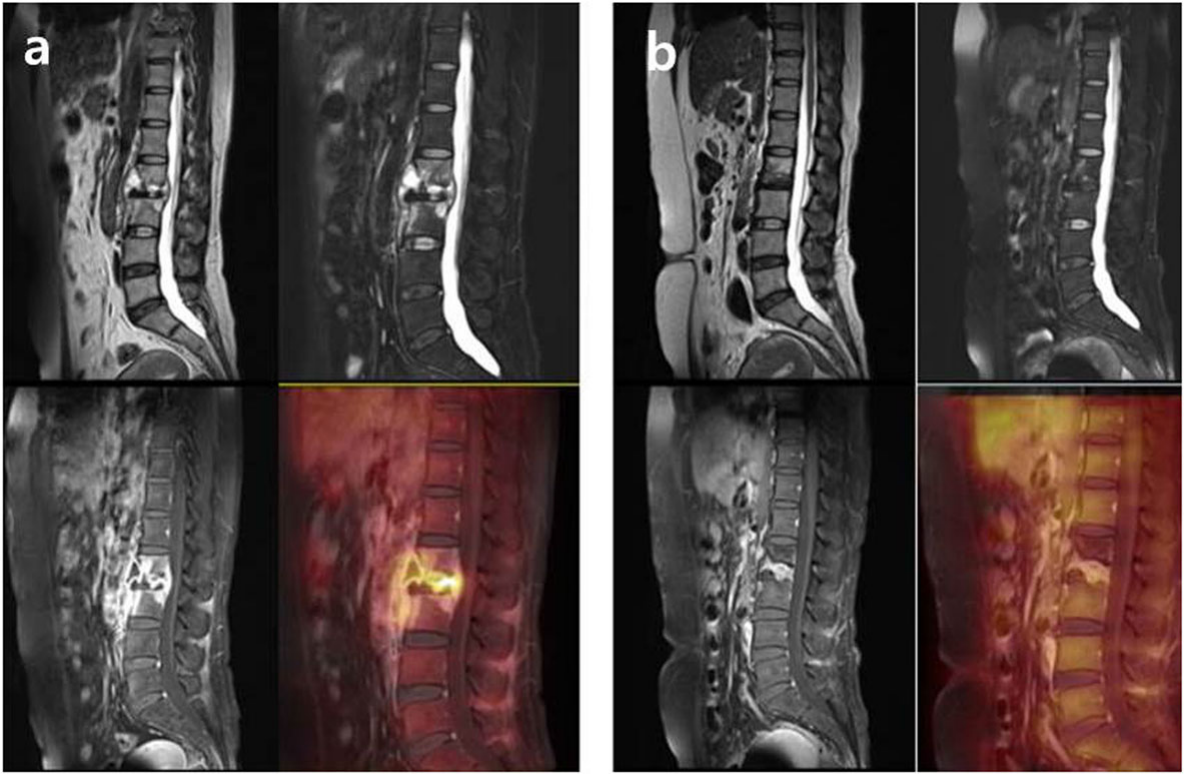
The first patient’s PET/MRI at 12-month anti-TB medication therapy (**b**) revealed the decreased bone marrow edema, pre- and intra-vertebral abscesses, paraspinal soft tissues, and SUV_max_ (from 9.75 to 1.83) on L2–3 compared with PET/MRI at 4-month anti-TB medication therapy (**a**). There was no relapse during the one-year follow-up period after discontinuation of treatment

Following the results of a percutaneous needle biopsy, positive acid-fast bacilli (AFB), polymerase chain reaction (PCR), and caseation necrosis were detected and finally confirmed as tuberculous spondylitis. Although there was a medical history of pulmonary TB, the sputum test showed negative result. A 12-month anti-TB medication therapy (daily isoniazid, rifampicin, pyrazinamide, and ethambutol for initial two-month, followed by a ten-month daily isoniazid, rifampicin, and ethambutol) was carried out without drug-susceptibility test (DST) under percutaneous biopsy due to favorable progress during the follow-up periods. PET/MRI at 12-month anti-TB medication therapy confirmed the loss of pre- and intra-vertebral abscesses with the decreased maximum standardized uptake value (SUV_max_, from 9.75 to 1.83) compared with PET/MRI at 4-month anti-TB medication therapy (Fig. 1). Clinical, hematological (CRP and ESR were normalized at 4-months anti-TB medication therapy), and radiological tests showed no relapse during the one-year follow-up period.

**Fig. 2:**
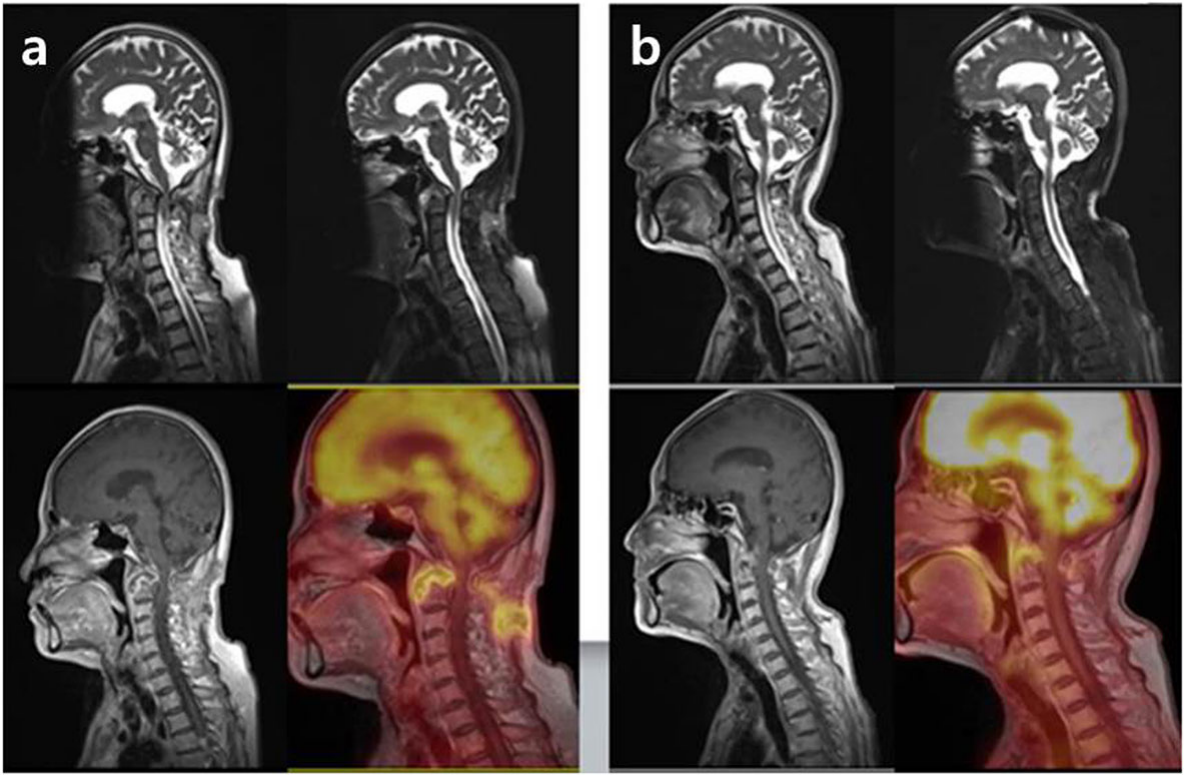
The second patient’s PET/MRI at 11.5-month anti-TB medication therapy (**b**) also revealed the decreased bone marrow edema with fatty change, pre- and intra-vertebral abscesses, and SUV_max_ (from 7.88 of PET/MRI at diagnosis to 4.14) on C1–2 compared with PET/MRI at diagnosis (**a**). However, positive AFB was detected from the granulation tissue of the surgical field, which resulted in additional anti-TB medication therapy

The second patient was a 64-year-old woman who presented with about six months of posterior neck pain. The patient had no previous TB history. MRI showed infectious spondylitis on C1–2 with a totally eroded odontoid process and paravertebral abscesses. CRP showed nearly normal range at diagnosis and during the whole treatment periods. ESR was increased by 71 mm/h and normalized at 3-month of anti-TB medication therapy. There was also the destruction of the left C1 and C2 bony structures with slight neck torticollis. Tuberculous spondylitis was confirmed with positive AFB, PCR, and caseation necrosis in an open biopsy. Surgical fixation and fusion were performed due to deterioration of the torticollis and instability of the cervical spine after 11.5-month of anti-TB medication therapy (daily isoniazid, rifampicin, pyrazinamide, and ethambutol for initial two-month, followed by a nine-months daily rifampicin and ethambutol due to resistance for isoniazid on the pus through the fistula of biopsy site). The territory of the lesion and SUV_max_ (from 7.88 to 4.14) had decreased on the PET/MRI conducted just before surgery compared with PET/MRI at diagnosis (Fig. 2). However, positive AFB and PCR were detected from the granulation tissue of the surgical field, which resulted in additional anti-TB medication therapy (added levofloxacin to rifampicin and ethambutol).

**Fig. 3:**
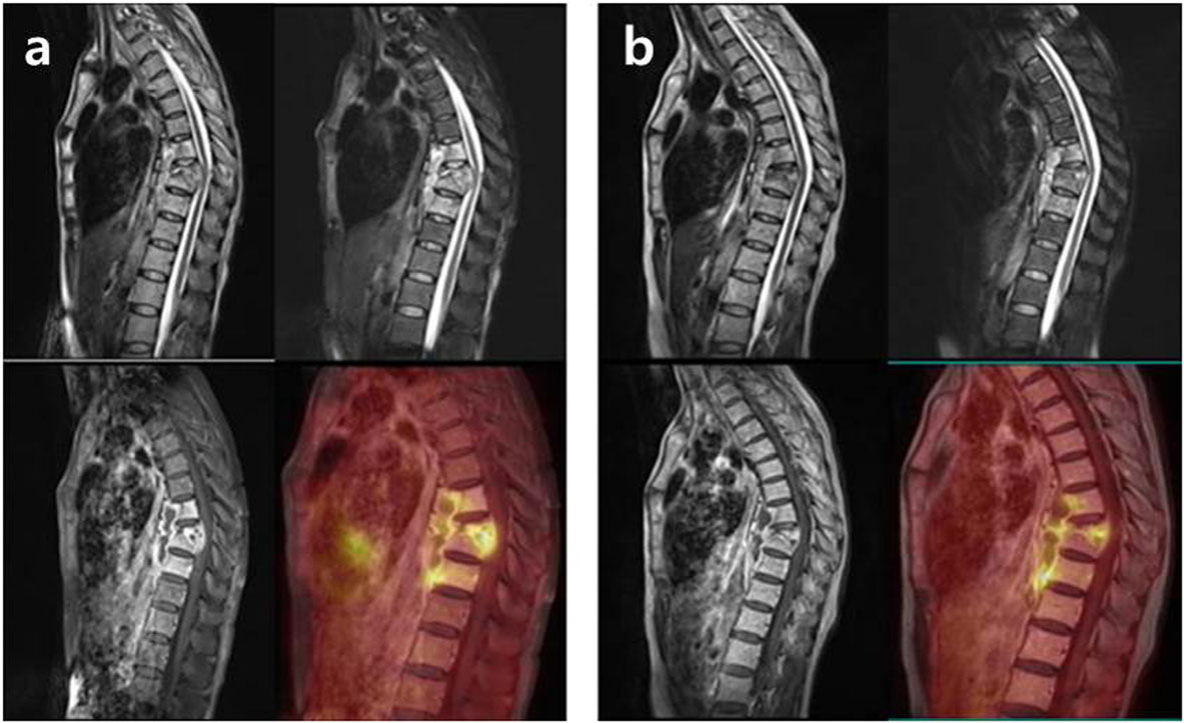
The third patient’s follow-up PET/MRI at 12-month anti-TB medication therapy (**b**) revealed sustained abscesses around the paravertebral space with intra-abscess SUV_max_ (from 6.67 of PET/MRI at diagnosis to 7.02) even though decreased bone marrow edema and epidural abscess on T8–9 compared with PET/MRI at diagnosis (**a**). After additional three months of anti-TB medication, the leak of the tuberculous abscess through the fistula developed again

The third patient is a 48-year-old woman who presented with about three months of back pain. CRP and ESR were 4.16 mg/dL and 115 mm/h, respectively (the indices have changed with the development and improvement of the fistula). Infectious spondylitis on T8–9 with paravertebral abscesses was confirmed on the MRI, and the result of the percutaneous needle biopsy showed positive AFB, PCR, and caseation necrosis, finally confirmed as tuberculous spondylitis. At the same time, active pulmonary TB was also confirmed and anti-TB medication therapy (daily isoniazid, rifampicin, pyrazinamide, and ethambutol for initial two-month, followed by a ten-month daily isoniazid, rifampicin, and ethambutol) started. Afterwards, a fistula was formed along the biopsy tract between deep lesion and skin, which resulted in repeated wound debridement and closure. Discontinuation of anti-TB medication therapy was considered after 12-month, because there were improvements in the pulmonary lesions and overall clinical features with healing of fistula. However, PET/MRI at 12-month anti-TB medication therapy showed sustained abscesses around the paravertebral space with intra-abscess SUV_max_ (from 6.67 to 7.02) even though decreased bone marrow edema and epidural abscess compared with PET/MRI at diagnosis (Fig. 3). After additional three months of anti-TB medication, the leak of the tuberculous abscess through the fistula developed again. However, there was no drug resistance on DST of the pus through the repeated fistula.

Culture-based phenotypic DST and GenoType MTBDR*plus* (a reverse hybridization line probe assay, Hain Lifesciences, Nehren Germany) are performed on the samples positive for M. *tuberculosis*. Phenotypic DST will be determined by the absolute concentration method using Lowenstein-Jensen media as recommended by WHO [7] at the supranational reference laboratory. The drugs and their critical concentrations for resistance in the patients are as follows: isoniazid 0.2 μg/mL, rifampin 40 μg/mL, and ethambutol 2.0 μg/mL. Pyrazinamide susceptibility was determined by a pyrazinamide test [8].

Clinical characteristics and assessment for therapeutic response of the patients are summarized in Table 1.

**Table 1:** Clinical characteristics and assessment for therapeutic response of the patients

	Patient 1	Patient 2	Patient 3
Clinical characteristics			
Age/Sex	43/Women	64/Women	48/Women
Initial ESR (mm/h)/CRP (mg/dL)	30/0.7	51/0.536	115/4.16
Lesion	Spondylitis on L2–3 Para- and intra-vertebral abscesses	Spondylitis on C1–2 Totally eroded odontoid process Para-vertebral abscesses	Spondylitis on T8–9 Para-vertebral abscesses
Drug-resistance	(−)	INH (+) on pus from fistula at biopsy site	(−)
HIV serology	(−)	(−)	(−)
SUV_max_ of PET/MRI 1	9.75 (at four-month of anti-TB medication)	7.88 (at diagnosis)	6.67 (at diagnosis)
Assessment for therapeutic response	Controlled Discontinuation of anti-TB medication	Uncontrolled Continuation of anti-TB medication	Uncontrolled Continuation of anti-TB medication
Timing	12-month after anti-TB medication (two-month with INH, RFP, PZA, and EMB/ ten-month with INH, RFP, and EMB)	11.5-month after anti-TB medication (two-month with INH, RFP, PZA, and EMB/ nine-month with RFP and EMB)	12-month after anti-TB medication (two-month with INH, RFP, PZA, and EMB/ ten-month with INH, RFP, and EMB)
Clinical state	Improved	Improved	Back pain Recurrent development of fistula at biopsy site
Evidence	Clinical improvement	AFB and PCR (+) on surgical biopsy	AFB and PCR (+) on pus from fistula
SUV_max_ of PET/MRI 2	1.83	4.14	7.02

ESR: erythrocyte sedimentation rate, CRP: C-reactive protein, HIV: human immunodeficiency virus, SUV_max_: maximum standardized uptake value, PET/MRI: ^18^F-fluorodeoxyglucose positron emission tomography/magnetic resonance imaging, TB: tuberculosis, INH: isoniazid, RFP: rifampin, PZA: pyrazinamide, EMB: ethambutol, AFB: acid-fast bacilli, PCR: polymerase chain reaction

## Discussion and conclusions

The definitive diagnosis of tuberculous spondylitis is based on the result of cultural and pathological tests conducted in tissues collected from lesions. However, this method is only found to be positive at 50–75%, and it is often necessary to conduct re-examination due to the high risk of false negatives [3, 9]. Sputum microscopy is available in pulmonary TB to determine treatment response, but it is not suitable in tuberculous spondylitis. Repeated biopsy and culture studies for detecting residual infectious lesion are neither reasonable nor perfect. Evaluation of therapeutic response in patients with tuberculous spondylitis TB usually indirectly depends on clinical and hematological features [6, 10, 11].

An MRI is a highly sensitive test, and it can also be used to determine therapeutic response. Recent study has defined a significant regression in the epidural or paraspinal abscess/granulation tissues, marrow reconversion, and fatty reconstitution of the diseased bone as features of radiological healing [12]. Le Page et al. reported gradual conversion of initial vertebral body edemas to fatty signals in 40% of cases at six months and 75% at 12 months [13]. However, the radiological findings of healing are a process of long-term imaginal changes without regard to clinical symptoms. Marrow edema and vertebral involvement can worsen by six months, and changes can even be observed at up to 14 months [14, 15]. MRIs cannot differentiate active lesion from sterile residual ones, and many patients continue to present pain due to several reasons even after they have been healed of TB [12]. Additionally, MRIs cannot reveal when anti-TB medication therapy should be discontinued. In the first patient, specific MRI features, including decrease in marrow edema, abscess size, and paraspinal soft tissues associated with the healing process were shown after 12 months of anti-TB medication therapy. In the second patient, however, TB existed continuously even though MRI features were related to the healing process.

For this reason, many efforts have been made to apply the 18F-FDG PET to evaluating therapeutic response, and several reports have recently been introduced. Using 18F-FDG PET measures the metabolic activity of the tissues in a non-invasive and semi-quantitative way, providing very accurate localization of the hypermetabolic activity. Active TB lesions are often proliferative lesions composed of epithelioid cells, Langerhans giant cells, and lymphocytes. These cells have high metabolisms of glucose and show a high uptake of FDG [16, 17]. Changes in FDG accumulation may be an important sign indicating the effect of anti-TB medication therapy. Metabolic responses may indicate therapeutic response and guide duration of antimicrobial therapy [18, 19].

From the results of the 18F-FDG PET in our patients, we expected the therapeutic response and completion of anti-TB medication therapy to accord with the SUV_max_. The first patient, whose treatment had been completed, showed SUV_max_ of 1.83, which is a very low as much as physiological FDG uptake in normal structures. In the second and third cases, SUV_max_ of 4.14 and 7.02, respectively, were found to be the higher uptake of SUV_max_ continuously within the paravertebral abscesses. These are judged to be a more accurate and consistent indication of the state of the TB lesion than MRI. The lesion presenting higher uptake of FDG can be explained by the overpopulation of the inflammatory cells described above, which are consistently present within the infected structures such as abscesses or vertebrae.

The optimal duration of anti-TB medication therapy is controversial. The Medical Research Council (MRC) advocates short-course chemotherapy (six months) for uncomplicated spinal TB [20]. However, Cormican et al. reported that mean duration of treatment was 13 months (range: 9–24 months) [21]. The region of the authors of this paper shows higher incidence and prevalence of drug resistance in TB. Therefore, the determination of the treatment duration should consider the regional characteristics and severity of the lesion, and discontinuation of anti-TB medication therapy should be prudent and conservative. When a relapse occurs, it usually happens within 12 months after the completion of therapy, indicating that the disease was incompletely treated [22].

There is a difference in the degree of FDG uptake depending on the presence of TB even after 12 months of anti-TB medication therapy. Especially in the third patient with active TB, the SUV_max_ (7.02) was kept very high regardless of the treatment duration, which meant that active TB was sustained and eventually led to abscess discharge through fistula again. However, as with the second patient, it was difficult to determine whether anti-TB medication should be discontinued, considering the healing process of the overall lesions and constantly reduced SUV_max_ (4.14). Successfully treated pyogenic spondylitis sometimes shows sustained high level of FDG uptake of 18F-FDG PET following weeks of antibiotics treatment, which can be explained by severe tissue damage and its healing process. Unlike pyogenic spondylitis, tuberculous spondylitis takes more time to sterilize than pyogenic spondylitis. The damaged tissues are also expected to be restored over long periods of sterilizing TB, resulting in lower FDG uptake (SUV_max_ 1.83 of the first patient) at the end of treatment than that of pyogenic spondylitis. Given this, the second patient can be determined to have a continuous TB and require further anti-TB medication therapy.

We think that 18F-FDG PET/MRI can be considered as a helpful independent and alternative method for determining the appropriate time to discontinue anti-TB medication. However, 18F-FDG PET/MRI is still very expensive examination and has low accessibility, which is limited to apply generally in the patients with TB spondylitis. Further studies with more patients are required to demonstrate our results and to overcome the limitations.

## Data Availability

The datasets used and/or analyzed during the current study are available from the corresponding authors on the reasonable request.

## Abbreviations

18F-FDG PET: Fluorine-18 fluorodeoxyglucose positron emission tomography
AFB: Acid-fast bacilli
PCR: Polymerase chain reaction
PET/MRI: Fluorine-18 fluorodeoxyglucose positron emission tomography /magnetic resonance imaging
SUVmax: Maximum standardized uptake value
TB: Tuberculosis
